# Genomic study of nonsyndromic hearing loss in unaffected individuals: Frequency of pathogenic and likely pathogenic variants in a Brazilian cohort of 2,097 genomes

**DOI:** 10.3389/fgene.2022.921324

**Published:** 2022-08-30

**Authors:** Caio Robledo D’ Angioli Costa Quaio, Antonio Victor Campos Coelho, Livia Maria Silva Moura, Rafael Lucas Muniz Guedes, Kelin Chen, Jose Ricardo Magliocco Ceroni, Renata Moldenhauer Minillo, Marcel Pinheiro Caraciolo, Rodrigo de Souza Reis, Bruna Mascaro Cordeiro de Azevedo, Maria Soares Nobrega, Anne Caroline Barbosa Teixeira, Matheus Martinelli Lima, Thamara Rayssa da Mota, Marina Cadena da Matta, Gabriela Borges Cherulli Colichio, Aline Lulho Roncalho, Ana Flavia Martinho Ferreira, Gabriela Pereira Campilongo, Eduardo Perrone, Luiza do Amaral Virmond, Carolina Araujo Moreno, Joana Rosa Marques Prota, Marina de França, Murilo Castro Cervato, Tatiana Ferreira de Almeida, Joao Bosco de Oliveira Filho

**Affiliations:** ^1^ Hospital Israelita Albert Einstein, São Paulo, SP, Brazil; ^2^ Instituto da Criança (Children’s Hospital), Hospital Das Clínicas HCFMUSP, Faculdade de Medicina da Universidade de São Paulo, São Paulo, Brazil; ^3^ VarsOmics, Sociedade Beneficente Israelita Brasileira Albert Einstein, São Paulo, SP, Brazil; ^4^ Programa de Pós Graduação em Tecnologias Energéticas e Nucleares (PROTEN), UFPE, Recife, Brazil; ^5^ Departamento de Morfologia e Genética, Universidade Federal de São Paulo, São Paulo, SP, Brazil; ^6^ Departamento de Medicina Translacional, Área de Genética Médica e Medicina Genômica, Faculdade de Ciências Médicas, Universidade Estadual de Campinas, Campinas, SP, Brazil

**Keywords:** nonsyndromic hearing loss, hearing loss, deafness, genomics, whole genome sequencing, GJB2 (C×26) gene mutations, STRC gene

## Abstract

Hearing loss (HL) is a common sensory deficit in humans and represents an important clinical and social burden. We studied whole-genome sequencing data of a cohort of 2,097 individuals from the Brazilian Rare Genomes Project who were unaffected by hearing loss to investigate pathogenic and likely pathogenic variants associated with nonsyndromic hearing loss (NSHL). We found relevant frequencies of individuals harboring these alterations: 222 heterozygotes (10.59%) for sequence variants, 54 heterozygotes (2.58%) for copy-number variants (CNV), and four homozygotes (0.19%) for sequence variants. The top five most frequent genes and their corresponding combined allelic frequencies (AF) were *GJB2* (AF = 1.57%), *STRC* (AF = 1%), *OTOA* (AF = 0.69%), *TMPRSS3* (AF = 0.41%), and *OTOF* (AF = 0.29%). The most frequent sequence variant was *GJB2*:c.35del (AF = 0.72%), followed by *OTOA*:p. (Glu787Ter) (AF = 0.61%), while the most recurrent CNV was a microdeletion of 57.9 kb involving the *STRC* gene (AF = 0.91%). An important fraction of these individuals (n = 104; 4.96%) presented variants associated with autosomal dominant forms of NSHL, which may imply the development of some hearing impairment in the future. Using data from the heterozygous individuals for recessive forms and the Hardy–Weinberg equation, we estimated the population frequency of affected individuals with autosomal recessive NSHL to be 1:2,222. Considering that the overall prevalence of HL in adults ranges from 4–15% worldwide, our data indicate that an important fraction of this condition may be associated with a monogenic origin and dominant inheritance.

## 1 Introduction

Hearing loss (HL) is the most common sensory deficit in humans. Congenital forms of HL may affect up to 1 in 500 newborns, and approximately 80% of prelingual deafness has a monogenic basis; on the other hand, age-related HL may affect up to 80% of individuals by the age of 80 and has a multifactorial basis in which cumulative noise exposure or other extrinsic damage along with individual genetic predisposition leads to impairment in cochlear transduction of acoustic signals (Yamasoba et al., 2013). Between these two extreme ages, several Mendelian forms of later-onset HL have been described. Overall, studies estimate that more than 360 million people worldwide may suffer the clinical and social burden of HL (Shearer & Smith, 2015).

HL may be classified as syndromic when presented with additional clinical manifestations involving other organs or systems or nonsyndromic when HL is the sole clinical finding. Nonsyndromic hearing loss (NSHL) comprises approximately 70–80% of genetic deafness. HL may also be clinically classified according to type, onset, severity, clinical progression, and acoustic frequency (Shearer et al., 2017).

There are important initiatives to map all genetic forms of HL, such as the Online Mendelian Inheritance in Man® (OMIM) database and the Hereditary Hearing Loss Homepage (G Van Camp & R J H Smith). However, studies with comprehensive genetic tests that include the great majority of known hearing impairment genes still leave over 50% of cases unsolved (Shearer and Smith, 2015), indicating that approximately half of the genetic forms of HL have yet to be elucidated.

Whole genome sequencing is likely the most unbiased method for the diagnosis of monogenic diseases because it does not involve a capture step targeting specific areas, thus providing a much broader net for molecular diagnoses. This method allows the study of sequence variants (including single-nucleotide variants [SNVs] and insertions–deletions [indels]) and copy-number variants (CNVs) not only in the coding regions of chromosomes but also in the great majority of noncoding regions and mitochondrial DNA (Lionel et al., 2018). Therefore, this powerful diagnostic tool may enrich the understanding of the underlying molecular mechanisms of the great majority of known monogenic forms of NSHL.

In this study, we aimed to assess pathogenic and likely pathogenic variants associated with NSHL in a cohort of unaffected Brazilian individuals referred for genome sequencing.

## 2 Materials and methods

### 2.1 Case selection: The Brazilian Rare Genomes Project

The Brazilian Rare Genomes Project is a public‒private partnership between Hospital Israelita Albert Einstein and the Brazilian Health Ministry that aims to offer large-scale genomic studies for patients with suspected rare diseases of genetic etiology. The project involves 16 public health institutions from nine states of Brazil.

In the current study, we preselected 2,199 patients who had been referred for molecular investigation with genome sequencing from 2020 to 2021. All clinical data were provided by the participating centers and were collected through a comprehensive pretest form filled in by the attending physician on the online platforms REDCap (Harris et al., 2009; Harris et al., 2019) and PhenoTips (Girdea et al., 2013); medical reports, clinical notes, and pictures of patients were provided to the laboratory in selected cases and were used to gather clinical data for this work if available. Patients were not evaluated by specialists from our team.

All clinical features were tabulated and categorized according to the Human Phenotype Ontology (HPO) database (Köhler et al., 2021). Participating patients were divided into two groups:

1) Patients with HL (HP:0000365, HP:0000399, HP:0000407, HP:0008527, HP:0008610, HP:0008619, HP:0008625, HP:0011476, HP:0011474, HP:0000405, HP:0040119, HP:0008513, HP:0012716, HP:0000408, HP:0008504, HP:0000410, HP:0001757, HP:0008573, HP:0008598, HP:0000365, HP:0008587, HP:0008596, HP:0005101, HP:0000364, HP:0008615, and HP:0009900) and
2) Patients without HL: all population assumptions and projections based on the data gathered in this study were based on this group of patients, considered “unaffected.”

### 2.2 Ethics statement

All patients or their legal guardians provided written consent before genome analysis. The project and related studies adhered to the Declaration of Helsinki principles for research on human beings and were granted ethics committee approval from all institutions involved (Plataforma Brasil; CAAE#29567220.4.1001.0071).

### 2.3 Molecular analysis and bioinformatics

Blood collection kits along with strict instructions for a blood draw and sample storage were sent to all participating centers. Peripheral blood samples were collected from all patients and shipped to our core facility in São Paulo, Brazil. DNA from the proband and, when available, from parents was immediately extracted using a QIAsymphony DNA Mini Kit and QIAsymphony automated system (Qiagen, Valencia, CA, United States); we used a NanoDrop 2000 (thresholds: 260/280 ratio ≈1.8 and 260/230 ratio between 1.8 and 2.2) and Qubit® 4 fluorometer using the Qubit® dsDNA HS assay (both Life Technologies, Carlsbad, CA, United States) for DNA quality assessment and quantification, respectively.

DNA fragmentation (Covaris ME220 ultrasonicator) and library preparation (Illumina TruSeq® DNA PCR-Free Library Prep protocol HS [Illumina Inc. San Diego, CA, United States]) strictly followed the best laboratory practices and instructions from the manufacturers. Sequencing was performed on an Illumina NovaSeq® 6000 platform. Complete details regarding genome sequencing steps and validation are in the preprint (Campos Coelho et al., 2021).

Genome data from every patient were aligned to the GRCh38/hg38 reference genome, and variants (SNVs, indels, and CNVs) were called using the DRAGEN Germline pipeline (Illumina, version 3.6.3 or superior); variants were annotated using in-house protocols and analyzed on the Varstation ® Platform (version 2.0, São Paulo, Brazil, www.varstation.com). Quality control (QC)-passing samples were those with a percentage of mapped reads >98%, autosome median coverage ≥20X, uniformity of coverage ≥80%, estimated cross-contamination level <2%, autosome callability ≥95%, percentage of bases that met Q30 scores (Q30 score) ≥ 90%, percentage of chimeric (supplementary) reads <5%, and percentage of mapped reads marked as duplicate <10%.

We assessed the evidence for consanguinity by including questions in the recruitment forms and quantifying the number of runs of homozygosity (NROH) with lengths ≥1 million base pairs (MB), the sum of all NROH lengths (SROH), and the frequency of ROH (FROH). The ROH coordinates were generated during alignment and variant calling using the DRAGEN Germline pipeline (Illumina, version 3.6.3 or superior; see Section 2.3 for more details). We calculated the NROH and SROH for each individual using scripts for R software version 4.1.3. We considered evidence for consanguinity if the individual’s SROH was greater than 123 MB; probable consanguinity ≥79 MB and ≤123 MB; probable nonconsanguinity <79 MB, and no evidence of consanguinity for ≤22 MB SROH (Matalonga et al., 2020).

### 2.4 Gene selection, gene-disease classification, and clinical impact

The list of genes associated with NSHL was defined based on the aggregated data available in 1) the Online Mendelian Inheritance in Man® (OMIM) database (Online Mendelian Inheritance in Man OMIM®, 2021); 2) a review by Shearer et al. (2017); 3) the Hereditary Hearing Loss Homepage (G Van Camp & R J H Smith); and 4) other available literature.

We used a systematic approach to collect available scientific evidence to qualitatively define the clinical validity of gene-disease associations for all genes, following the framework proposed by the Clinical Genome Resource—ClinGen (Strande et al., 2017). The gene-disease classifications proposed by ClinGen and adopted in this study are as follows: limited, moderate, strong, and definite; for contradictory evidence, genes were classified as disputed and refuted. Several genes studied in our work had already been classified by the ClinGen Gene Curation Working Group and were used as standards for these genes. For the remaining genes not previously classified by ClinGen, our team replicated the ClinGen approach to curate the gene-disease validity.

Using data available in OMIM and the literature, we reviewed the following molecular and clinical characteristics for every gene: inheritance pattern, predominant age of onset of hearing impairment (prelingual or postlingual), type of HL (conductive, sensorineural, mixed, and central), predominant severity (mild: 26–40 dB, moderate: 41–70 dB, severe 71–90 dB, and profound >90 dB), clinical progression (progressive and stable), and predominantly affected frequencies (low: <500 Hz, middle: 501–2000 Hz, and high: >2000 Hz). For the purpose of this study, we grouped moderate (41–55 dB) and moderately severe (56–70 dB) HL into a single group of moderate HL (41–70 dB).

### 2.5 Variant preselection and classification

We used an in-house script based on Python 3.8 to search for variants of potential clinical interest in genes associated with NSHL for all patients using MANE and RefSeq transcript coordinates as references; we shared this script code in the GITHUB repository at the following address: https://github.com/Varstation/non-syndromic_hearing_loss. These variants were searched in variant call format (VCF) files for every patient according to the following selection criteria:

1) For SNVs and small indels, we preselected all variants previously reported in the ClinVar database that were classified as pathogenic, likely pathogenic, or variants that had a conflicting pathogenicity classification for every patient; variants reported in ClinVar as benign or likely benign were excluded from this preselection process.
2) For CNVs, all copy-number variants (microdeletions or microduplications) that included at least one base pair harbored by coding regions were preselected using AnnotSV (v.2.5) (Geoffroy et al., 2018; Geoffroy et al., 2021); CNVs encompassing solely noncoding regions were not preselected. All CNVs were visually confirmed in BAM files using the Integrative Genomics Viewer (IGV) tool (Robinson et al., 2011).
3) Smaller structural variants (SVs) were also selected using a different approach: unbalanced SVs (microdeletions and microduplications, which may be considered small CNVs), affecting whole genes or predicted leading to frameshift as indicated by AnnotSV. This approach was also used because detecting small CNVs may be a more challenging task and complements the previous step. All unbalanced SVs were visually confirmed in BAM files using IGV. In this study, unbalanced SVs (small CNVs) and CNVs were considered in the same group of CNVs.
4) For mitochondrial variants, only selected variants already associated with HL were searched (Lott et al., 2013).

Regarding sequence variants, we excluded from our analysis 1) variants not present in ClinVar, 2) variants with insufficient read coverage (less than 20X), and 3) variants with a call rate <95% considering the whole sample. For CNVs, we excluded events potentially associated with syndromic conditions: 1) CNVs that involve critical regions for known forms or contiguous-gene microdeletion or microduplication syndromes cataloged by ClinGen (including data from the International Standards for Cytogenomic Arrays—ISCA— Consortium) and 2) CNVs larger than 1 Mb (one megabase).

The complete list of searched nuclear genes is shown in Supplementary Table S1, and the list of mitochondrial variants is shown in Supplementary Table S2.

Sequence variants were classified according to ACMG guidelines (Richards et al., 2015) incorporating later ClinGen working group recommendations. CNVs were classified following the standards of the joint consensus recommendation for the interpretation and reporting of constitutional copy number variants of the American College of Medical Genetics and Genomics (ACMG) and ClinGen (Riggs et al., 2020). For the classification of CNVs, we used the ClinGen web-based CNV classification calculator (cnvcalc.clinicalgenome.org/cnvcalc/).

### 2.6 Homozygous genotype frequency estimation

We used the Hardy–Weinberg equation to estimate variant homozygous genotype frequencies (q^2^) considering the respective carrier frequencies (2pq) observed in this study (Edwards and Hardy, 2008). For this purpose, we considered random mating and the approximation p∼1.

### 2.7 Statistical analysis

We calculated allele counts and frequencies by directly counting genotypes extracted from filtered VCF files using Unix Bash and R software version 4.1 scripts. Next, we verified which ClinVar variants detected in our sample were also detected in the Brazilian ABraOM cohort (Naslavsky et al., 2017). Then, we compared the allele counts using a two-tailed Fisher exact test to assess whether there were significant differences in allele distribution between the samples. The resulting *p-*values were adjusted using the false discovery rate (FDR) method for multiple comparisons, and the significance level was set at α = 0.05.

## 3 Results

### 3.1 Selected cases

**FIGURE 1:**
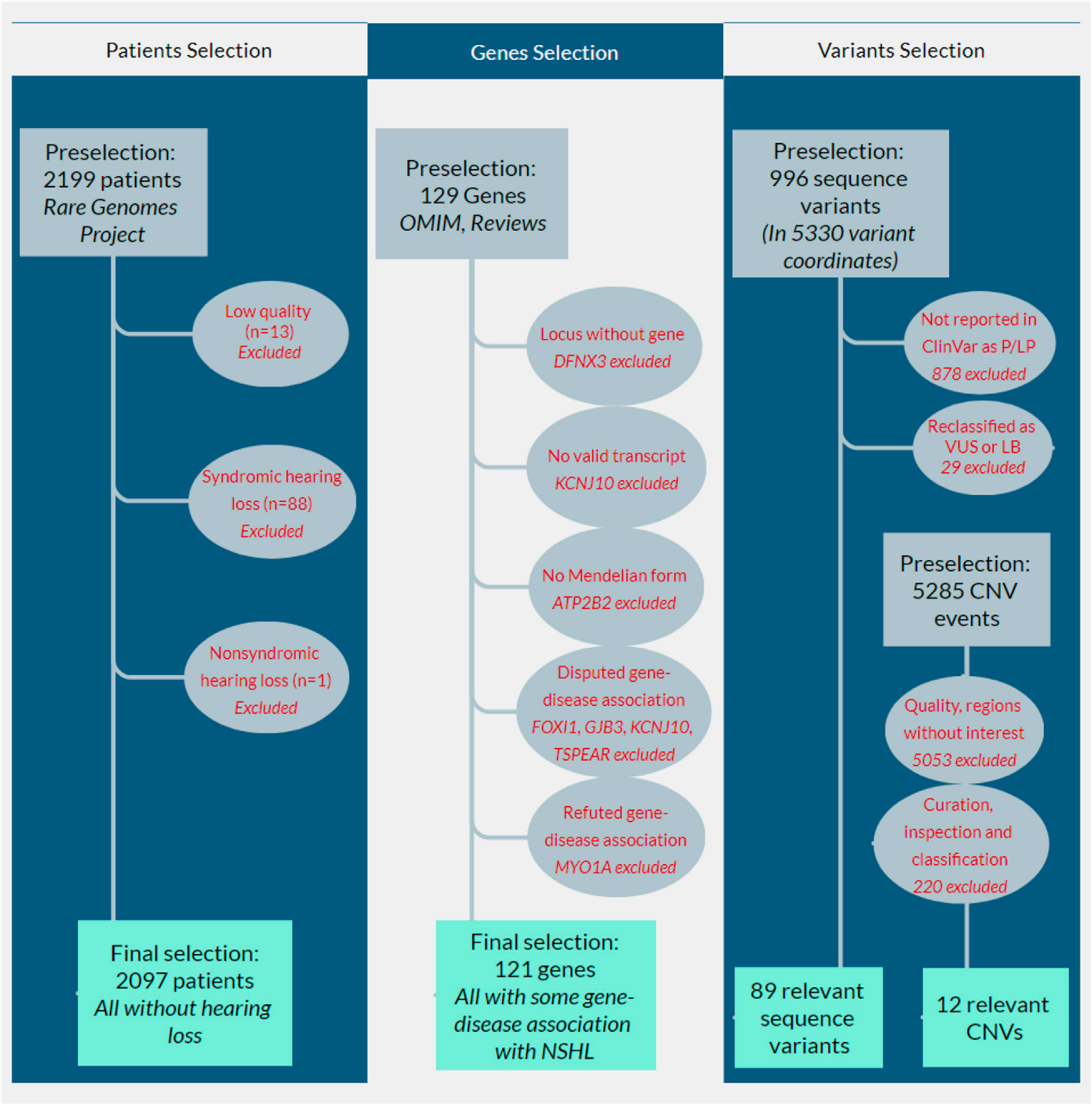
Flowcharts showing the selection workflow for patients, genes, and variants. The first column shows the patient selection workflow: 2,199 patients who had been referred for molecular investigation using whole genome sequencing from 2020–2021 were preselected; 13 failed sample quality controls, and 89 patients presented with hearing loss (88 syndromic hearing loss and one with nonsyndromic hearing loss [NSHL]): all were excluded from our cohort and population calculations. The second column shows gene selection: 129 genes associated with NSHL were preselected from the OMIM database, reviews (Shearer et al., 2017; Van Camp and Smith, 2021), and other available literature; three of them were initially excluded because one of them (*DFNX3*) refers to a locus without a known gene, another (*KCNJ10*) did not present a valid transcript in MANE or RefSeq databases, and the other (*ATP2B2*) was not associated with a Mendelian form of hearing loss; four genes (*FOXI1*, *GJB3*, *KCNJ10*, and *TSPEAR*) were excluded for presenting disputed gene-disease association and another gene (*MYO1A*) refuted association. The third column shows the variant selection workflow: 1) for sequence variants, 996 variants were preselected in 5,330 variant coordinates, but only 118 were reported in ClinVar as pathogenic or likely pathogenic (P/LP); the remaining 878 were excluded; after our internal curation and reclassification, 29 variants were excluded because they were reclassified as variants of unknown significance (VUS, n = 28) or likely benign (LB, *n* = 1); 2) for copy-number variants (CNV), the filtering process of 5,285 preselected CNV events eliminated 5,053 CNVs; our internal curation, visual inspection, and classification eliminated another 220.

Figure 1 shows the workflow for selecting patients, genes, and genetic variants for downstream analysis. Among the 2,199 preselected patients, 13 failed the sample QC (mostly due to median autosome coverage <20X) and were removed from the analysis. Among the remaining 2,186, 89 patients presented with HL: 88 (98.9%) presented with other systemic involvement, such as neurological manifestations (69/88, 78.4%) or growth anomalies (60/88, 68.2%) and were clinically classified as having a syndromic form of HL; only one patient (1.1%) presented isolated (e.g., nonsyndromic) HL; all 89 patients were excluded from our cohort and population calculations.

Therefore, 2,097 patients did not present any form of HL and were included in our definite cohort of unaffected individuals. All data, calculations, and estimates in this work refer to this group of individuals.

Self-reported information regarding consanguinity was available for 1,615 individuals (77.0%). Among those, 1,424 (67.9%) reported no history of known consanguinity in the family, whereas 191 (9.1%) provided positive indications for consanguinity in the family. SROH and NROH were available for 2,069 individuals (98.7%). Mean overall NROH = 5.3 ± 6.6, mean SROH = 30.3 ± 76.4 MB, and mean FROH = 1.1% ± 2.7%. Self-report did not always match the classification using the ROH metrics. Our preliminary data suggest that approximately 8.0% (data not shown) of individuals had a mismatch between self-report and SROH data. Among patients with more than 79 MB following Matalonga et al.’s (2020) classification (n = 221, 10.5%), mean NROH = 20.5 ± 9.7, SROH = 213.2 ± 127.1 MB, and FROH = 7.4% ± 4.4%. Therefore, our sample seems to have a minority of individuals who likely have consanguineous parents, and they present remarkable variation in their ROH metrics.

### 3.2 Disease-gene classification and clinical characteristics

We preselected a total of 129 genes of known association with NSHL; three of them were initially excluded from our evaluation because one of them (*DFNX3*) refers to a locus without a known gene, another (*KCNJ10*) did not contain a valid transcript in the MANE or RefSeq databases, and the other (*ATP2B2*) was not associated with a Mendelian form of HL (classified as a modifier by OMIM). We also eliminated four genes (*FOXI1*, *GJB3*, *KCNJ10*, and *TSPEAR*) with gene-disease associations classified as disputed by the ClinGen team and another gene (*MYO1A*) classified as refuted. Additionally, we eliminated the autosomal recessive (AR) form of *GJB6*, classified as refuted by ClinGen, keeping only its autosomal dominant (AD) form. The complete list of the seven excluded genes, evidence, and other details are available in Supplementary Table S3.

The remaining 121 genes (including the AD form of *GJB6*) were considered to present at least a minimal level of gene-disease association with NSHL and were individually curated. Considering these 121 genes, 42 present exclusively AD inheritance, 65 exclusively AR, six X-linked (XL), and eight present both AD and AR inheritances.

Regarding the 42 genes presenting exclusively AD inheritance, 27 (64.3%) presented a ClinGen gene-disease curation, and the remaining genes (n = 15, 35.7%) were curated by our group. Overall, the gene-disease association was classified as definite for 26.2% (n = 11), strong for 4.8% (*n* = 2), moderate for 19% (*n* = 8), and limited for 50% (n = 21). The great majority of AD inheritance genes (n = 35, 83.3%) were associated with predominantly postlingual-onset HL, while seven genes (16.7%; *CRYM*, *GREB1L*, *KITLG*, *PLS1*, *SIX1*, *SLC12A2*, and *WFS1*) were associated with prelingual onset. Progressive involvement was observed for the majority of these genes (85.7%, n = 36); the remaining genes presented a predominantly stable (7.1%, n = 3) or variable progression (4.8%, n = 2); for one gene (2.4%; *GREB1L*), progression was not clear in the literature. High and middle-high frequencies were predominantly affected in 54.8% (n = 23), followed by flatter involvement for all frequencies in 28.6% (n = 12), and for 16.7% (n = 7), the involvement of low, low-middle, or middle frequencies was more predominant.

For the 65 genes with exclusively AR inheritance, the great majority (55, 84.6%) presented a ClinGen gene-disease curation, and the remaining (n = 10, 15.4%) were curated by our group. Gene-disease association was classified as definite for 41.5% (*n* = 27), strong for 7.7% (n = 5), moderate for 15.4% (n = 10), and limited for 35.4% (n = 23). In contrast to AD forms, the majority of genes associated with AR HL (84.6%, n = 55) were associated with the predominant prelingual onset and 15.4% (n = 10) with postlingual onset. A stable hearing impairment was observed for the majority of these genes (58.5%, n = 38); the remaining presented a predominantly progressive (29.2%, n = 19) or variable progression (1.5%, n = 1; *CDC14A*); for seven genes (10.8%; *ESRP1*, *GAB1*, *GRAP*, *MET*, *SPNS2*, *TMEM132E*, and *WHRN*), the progression was not clear in the literature. A flatter pattern involving all frequencies was predominant in 56.9% (n = 37), while predominant high or middle-high frequencies were observed in 18.5% (n = 12) and middle frequencies in 1.5% (n = 1; *GRXCR1*), while for 23% (n = 15), a predominant frequency was not clear in the literature. A minority of AR forms were associated with mild-moderate HL (6.1%, n = 4), and the majority involved more severe phenotypes to varying degrees between moderate and profound involvement (93.8%, n = 61).

A minority of six genes presented XL inheritance, four (66.7%) of which presented ClinGen gene-disease curation. Half of the X chromosome genes presented gene-disease curation classified as definite (*AIFM1*, *POU3F4*, and *SMPX*), and the other half had limited evidence (*COL4A6*, *GPRASP2*, and *PRPS1*). Two genes (33.3%; *AIFM1* and *SMPX*) presented postlingual onset and the remaining prelingual onset. All genes were associated with progressive involvement, a flatter pattern involving all frequencies and generally moderate to profound manifestation.

The remaining eight genes (*COL11A2*, *GJB2*, *MYO6*, *MYO7A*, *PTPRQ*, *TBC1D24*, *TECTA*, and *TMC1*) were associated with both AD and AR inheritance. Except for *MYO7A* (AR) and *PTPRQ* (AD), ClinGen gene-disease curation was available for all remaining forms of all genes. For the *TECTA* gene, both AD and AR modes are associated with the prelingual onset; for all remaining genes, AD forms are associated with the postlingual onset and milder phenotypes, while AR forms are associated with the prelingual onset and more severe HL.

Table 1 shows a summary of the 121 genes associated with NSHL, inheritance pattern, predominant age of onset, and predominant frequencies. The complete list of genes studied in this work along with individual gene-disease classification and the resources used in this curation are available in Supplementary Table S1.

**TABLE 1:** Summary of genes involved in nonsyndromic hearing loss categorized by inheritance, predominant sound frequency impairment, and onset.

Inheritance	Predominant frequency	Onset	
		Prelingual	Postlingual
AD (n = 42 genes)	All	4	8
	High	2	17
	Middle-high	0	4
	Middle	0	3
	Low-middle	0	1
	Low	1	2
AR (n = 65 genes)	All	34	3
	High	5	4
	Middle-high	2	1
	Middle	1	0
	Not reported	13	2
AD/AR (n = 8 genes)^†^	All	6	1
	High	0	4
	Middle-high	0	0
	Middle	2	1
	Not reported	1	1
XL (n = 6 genes)	All	4	2
	Associations, subtotal	75	54
	Associations, total	129	
	Unique genes^†^	121	

Inheritance: AD, autosomal dominant; AR, autosomal recessive, XL, X-linked.

Frequency: low - < 500 Hz, middle - 501–2000 Hz, and high - > 2000 Hz.

^†^Eight genes (*COL11A2*, *GJB2*, *MYO6*, *MYO7A*, *PTPRQ*, *TBC1D24*, *TECTA*, and *TMC1*) are associated with both autosomal dominant and autosomal recessive forms. For the *TECTA*, gene, both AD and AR forms are associated with prelingual onset; for all remaining genes, AD forms are associated with postlingual onset, and AR forms are associated with prelingual onset.

### 3.3 Sequence variants

Initially, a total of 5,330 variant coordinates dispersed throughout the 121 nuclear genes and 23 mitochondrial genes (5099 autosomal, 119 on the X chromosome, and 112 mitochondrial) were preselected according to the criteria mentioned previously. Following variant read depth quality control (QC), the genotypes from 5,087 autosomal, 104 on the X chromosome, and all 112 mitochondrial coordinates were retrieved. We did not detect any mitochondrial variants from the list of variants associated with HL.

Following the sample, variant, and call rate QC, 996 variants were detected in either of the two study groups (993 autosomal and three on the X chromosome). Among these variants, a total of 118 variants were reported in the ClinVar database and have been classified as pathogenic or likely pathogenic (P/LP) by at least one submitter. These variants were then individually curated and classified according to the ACMG criteria by our team of geneticists: 29 variants were excluded because they were reclassified as variants of unknown significance (n = 28) or likely benign (n = 1).

Finally, we retained a total of 89 clinically relevant sequence variants associated with nonsyndromic HL that were detected in the unaffected patients. They were distributed among 36 distinct genes; 44 variants were classified as pathogenic and 45 as likely pathogenic according to ACMG criteria. All variants were harbored by autosomal genes; no variants on the X chromosome were classified as P/LP. The complete list of variants, details on nomenclature, and respective frequencies are available in Supplementary Tables S4 and 5 (genotypes per variant) (genotypes per gene). The list of excluded variants along with all molecular data and criteria used for final classification is available in Supplementary Table S6.

We identified 222 heterozygotes (10.6%) and four homozygotes (0.2%) for these 89 P/LP variants in the group of 2,097 patients without HL. The top six most frequent variants in heterozygosity and the absolute number of heterozygous and allelic frequencies (AF) are as follows:

1) *GJB2*(NM_004004.6): c.35del; p. (Gly12fs); n = 28 (AF = 0.72%)
2) *OTOA*(NM_144672.4): c.2359G>T; p. (Glu787Ter); n = 25 (AF = 0.61%)
3) *GJB2*(NM_004004.6): c.109G>A; p. (Val37Ile); n = 23 (AF = 0.55%)
4) *TMPRSS3*(NM_001256317.3): c.1273G>A; p. (Ala425Thr); n = 13 (AF = 0.31%)
5) MPZL2(NM_005797.4): c.72del; p. (Ile24fs); n = 8 (AF = 0.19%)
6) *GJB2*(NM_004004.6): c.617A>G; p. (Asn206Ser); n = 6 (AF = 0.14%)

Among the heterozygotes, the majority (n = 123, 55.4%) were carriers for P/LP variants in genes associated exclusively with AR inheritance; 89 (40.1%) presented variants harbored by genes associated with both AD and AR forms. The remaining 10 (4.5%) harbored monoallelic P/LP variants for five genes associated exclusively with AD forms of HL (genes *GJB6*, *KCNQ4*, *POU4F3*, *TJP2*, and *WFS1*).

Regarding the four homozygotes, each had a different variant in homozygosity: 1) *GJB2*(NM_004004.6): c.35del; p. (Gly12fs) (AF = 0.72%); 2) *DCDC2*(NM_016356.5): c.383C>G; p. (Ser128Ter) (AF = 0.07%); 3) *TBC1D24*(NM_001199107.2): c.724C>T; p. (Arg242Cys) (AF = 0.07%); and 4) *TJP2*(NM_004817.4): c.1234C>T; p. (Arg412Ter) (AF = 0.05%). The *TJP2* gene is exclusively associated with autosomal dominant inheritance, *DCDC2* exclusively with autosomal recessive inheritance, and the remaining two with both forms of inheritance.

The combined frequencies for all the different P/LP variants for the top ten most frequent genes are shown in Table 2.

**TABLE 2:** Summarized clinical and molecular data regarding pathogenic and likely pathogenic (P/LP) variants for the 10 genes with the highest heterozygous genotype frequency. Three genes are associated with both autosomal dominant and autosomal recessive (AD/AR) forms of inheritance, and clinical features regarding both inheritance mechanisms are separated by “/” if they differ; the number of unique variants, combined allele frequencies for P/LP variants, number of heterozygotes, and number of homozygotes are shown for sequence variants, copy-number variants (CNVs), and combined molecular mechanisms (sequence variants plus CNVs). Additionally, see Supplementary Tables S4 and S7 for details of the variants.

Genes	Mode of inheritance	Gene-disease association validity	Clinical features					Sequence variants				Copy-number variants				Combined molecular mechanisms			
			Onset	Type	Predominant severity	Progression	Predominant frequency	Number of unique P/LP variants (%)	Combined allele frequency	Heterozygotes	Homozygotes	Number of unique P/LP variants (%)	Combined allele frequency	Heterozygotes	Homozygotes	Number of unique P/LP variants (%)	Combined allele frequency	Heterozygotes	Homozygotes
*GJB2*	AD/ AR	Definitive/ definitive	Postlingual/ prelingual	Sensorineural	Moderate to severe/ profound	Progressive/ stable	High/ all	9 (10.1)	0.0157	64	1	0 (0.0)	0.0000	0	0	9 (8.9)	0.0157	64	1
*STRC*	AR	Definitive	Prelingual	Sensorineural	Moderate to severe	Stable	All	1 (1.1)	0.0007	3	0	2 (16.7)	0.0093	39	0	2 (2.0)	0.0100	42	0
*OTOA*	AR	Definitive	Prelingual	Sensorineural	Severe to profound	Stable	All	1 (1.1)	0.0060	25	0	1 (8.3)	0.0010	4	0	2 (2.0)	0.0069	29	0
*TMPRSS3*	AR	Definitive	Postlingual	Sensorineural	Severe to profound	Stable	Not reported	4 (4.5)	0.0041	17	0	0 (0.0)	0.0000	0	0	4 (4.0)	0.0041	17	0
*OTOF*	AR	Definitive	Prelingual	Sensorineural	Severe to profound	Stable	Middle-high	7 (7.9)	0.0029	12	0	0 (0.0)	0.0000	0	0	7 (6.9)	0.0029	12	0
*MYO7A*	AD/ AR	Definitive/ limited	Postlingual/ prelingual	Sensorineural	Moderate/ profound	Not reported/ Progressive	Not reported/ all	7 (7.9)	0.0021	9	0	0 (0.0)	0.0000	0	0	7 (6.9)	0.0021	9	0
*SLC26A4*	AR	Strong	Prelingual	Mixed	Moderate	Progressive	high	8 (9.0)	0.0021	9	0	0 (0.0)	0.0000	0	0	8 (7.9)	0.0021	9	0
*MPZL2*	AR	Strong	Prelingual	Sensorineural	Moderate to severe	Progressive	High	1 (1.1)	0.0019	8	0	0 (0.0)	0.0000	0	0	1 (1.0)	0.0019	8	0
*COL11A2*	AD/ AR	Moderate/- moderate	Postlingual/ prelingual	Sensorineural	Moderate to Severe/ severe to Profound	Stable/ stable	Middle/ all	2 (2.2)	0.0017	7	0	0 (0.0)	0.0000	0	0	2 (2.0)	0.0017	7	0
*PDZD7*	AR	Definitive	Postlingual	Sensorineural	Moderate to severe	Progressive	All	4 (4.5)	0.0017	7	0	0 (0.0)	0.0000	0	0	4 (4.0)	0.0017	7	0
							All other genes (n = 111)	45 (50.6)	0.0160	61	3	9 (75.0)	0.0026	11	0	54 (53.5)	0.0186	72	3
							All genes (n = 121)	89 (100.0)	0.0548	222	4	12 (100.0)	0.0129	54	0	101 (100.0)	0.0677	276	4

Here, thirteen of the 89 P/LP variants detected in the no HL group were also detected in the Brazilian ABRaOM study. We compared the distribution of allele counts between their cohort and ours using Fisher’s exact test. After correction for multiple comparisons, the differences in allelic counts were not statistically significant for 12 of them. Only *OTOA* (NM_144,672.4): c.2359G>T; p. (Glu787Ter) appeared to be less frequent in our sample when compared to that observed by ABraOM (FDR adjusted Fisher’s exact test *p*-value column in Supplementary Table S4).

### 3.4 Copy-number variants

A preselection of 5,285 CNV events affecting 1,757 independent gene loci was detected among 1,259 patients (51 with HL, 1,208 without HL). This initial group of CNVs underwent a filtering process to eliminate all events harbored by patients with HL and all CNVs restricted to noncoding regions, after which 213 CNVs and 19 structural variants (small CNVs) remained. All 232 alterations underwent curation, visual inspection in BAM files, and classification according to the joint recommendation of ACMG and ClinGen (Riggs et al., 2020).

Finally, a total of 12 different heterozygous CNVs in 54 individuals were considered clinically relevant: four were classified as pathogenic and eight were classified as likely pathogenic (Supplementary Table S7). The most recurrent pathogenic CNV observed in our cohort and present in heterozygosity in 38 individuals (AF = 0.91%) was a microdeletion of approximately 57.9 kb (coordinates: chr15:43600849_43658715) involving exons 1 through 26 (out of a total of 29 exons) of the *STRC* gene, associated with the AR form of NSHL, along with the *CATSPER2* gene (not associated with any Mendelian disorder in the OMIM database; however, biallelic deletions concomitantly involving *STRC* and *CATSPER2* are associated with HL and male infertility, OMIM:611102). Another individual had a nonrecurring microdeletion also involving *STRC* (46.6 Kb; exons 22 through 29 and the *PPIP5K1* gene; chr15:43557296_43603898; n = 1; AF = 0.024%).

The second-most recurrent pathogenic CNV was a microdeletion of approximately 244.2 Kb present in heterozygosity in four individuals (AF = 0.095%) involving the *OTOA* gene (associated with the AR form of NSHL) and the surrounding *METTL9* gene (not associated with any Mendelian disorder in OMIM). The other CNVs identified all in heterozygosity in our work are as follows:

1) Pathogenic microdeletion of 805.5 Kb (chr16:15394361_16199828) involving the *ABCC1* gene (AD form of NSHL): n = 2, AF = 0.048%;
2) Likely pathogenic microdeletion of 10.4 Kb (chr11:78476555_78486991) involving exons 7 through 10 of the *NARS2* gene (AR form of NSHL): n = 2; AF = 0.048%;
3) Pathogenic microdeletion of 400.5 Kb (chr17:18624545_19025057) involving the *GRAP* gene (AR form of NSHL): n = 1; AF = 0.024%;
4) Likely pathogenic microdeletion of 19.6 Kb (chr15:51437517_51457141) involving exons 36 through 43 of the *DMXL2* gene (AD form of NSHL): n = 1; AF = 0.024%;
5) Likely pathogenic microdeletion of 17.7 Kb (chr7:24702446_24720157) involving exons 3 through 8 of the *GSDME* gene (AD form of NSHL): n = 1; AF = 0.024%;
6) Likely pathogenic microdeletion of 10 kb (chr11:17603104_17613104) involving exons 33 through 38 of the *OTOG* gene (AR form of NSHL): n = 1; AF = 0.024%;
7) Likely pathogenic microduplication of 10.9 Kb (chr12:80288802_80299740) involving exon 27 of the *OTOGL* gene (AR form of NSHL): n = 1; AF = 0.024%;
8) Likely pathogenic microdeletion of 11.8 Kb (chr22:37719702_37731543) involving exon 5 of the *TRIOBP* gene (AR form of NSHL): n = 1; AF = 0.024%;
9) Intragenic likely pathogenic microdeletion of 11.6 Kb (chr11:121163035_121174624) involving exons 16 through 19 of the *TECTA* gene (AR/AD form of NSHL): n = 1; AF = 0.024%.

CNVs excluded for being of unknown significance or syndromic are shown in Supplementary Table S8.

### 3.5 Recessive HL frequency estimation

We used the Hardy–Weinberg equation to roughly estimate autosomal recessive HL frequency in our population (q^2^) based on the respective carrier frequencies (2pq) for all P/LP sequence variants and CNVs and the approximation of p∼1. Only genes associated with AR or both AD/AR forms were included in this estimate. According to this method, we estimated the following top five frequencies of autosomal recessive NSHL per gene per 1,000 people: *GJB2*: 0.25; *STRC*: 0.1; *OTOA*: 0.05; *TMPRSS3*: 0.02, and *OTOF*: 0.01. The combined frequency of these five recessive forms of NSHL is estimated to be 0.42 per 1,000 people, while the combined frequency considering all recessive forms of NSHL is 0.45/1,000 or 1:2,222. Supplementary Table S9 shows the estimate for each pathogenic/likely pathogenic variant included in this specific analysis.

## 4 Discussion

Herein, we studied molecular alterations (including sequence variants, copy-number variants, and mitochondrial DNA variants) associated with NSHL using data from genome sequencing of a cohort of 2,097 unaffected patients. We identified 276 heterozygotes (13.16%) and four homozygotes (0.2%) for P/LP variants harbored by genes associated with NSHL. Among the heterozygotes, 222 individuals (10.59%) were heterozygotes for 89 P/LP sequence variants and 54 (2.58%) were heterozygotes for 13 P/LP copy-number variants. All four homozygotes harbored P/LP sequence variants.

An important fraction of our cohort (n = 104, 4.96%) of unaffected individuals presented P/LP variants in genes associated with either exclusively AD or both AR/AD forms of NSHL. This finding may imply that at least a fraction of these individuals present mild hearing impairment or may develop it in the future, especially if we consider that 1) AD genes manifest primarily as postlingual onset (some forms in later decades of life), have progressive involvement, and predominantly affect high and middle-high frequencies and 2) the AD form of AR/AD genes is also associated with postlingual onset and milder phenotypes (Table 1). Considering that epidemiological studies have shown that the overall prevalence of HL in adults ranges from 4–15% worldwide (Hoffman et al., 2017; Sheffield and Smith, 2019), our data indicate that an important fraction of this condition may be associated with monogenic origin and dominant inheritance.

Among these 104 heterozygotes for dominant alleles for NSHL, the majority (61.5%, n = 64; 3.05% of the entire cohort) harbored monoallelic pathogenic variants in the *GJB2* gene. This gene is associated with both AR and AD forms of NSHL. Although a full penetrance and progressive course have been described for both forms of this gene, the clinical impact may be heterogeneous, varying from mild to severe and primarily affecting the perception of high frequencies (Smith and Ranum, 1993). The combined allele frequency of 1.41% for the top three most frequent variants in *GJB2* (c.35del, p.Val37Ile, and p.Asn206Ser) observed in our study (0.72, 0.55, and 0.14%, respectively) is close to the 1.05% observed in gnomAD (0.63, 0.4, and 0.02%) and the 1.49% in ABraOM (0.98, 0.34, and 0.17%). This gene is followed in the number of heterozygotes by *MYO7A* (n = 9; 0.43% of the cohort) and *COL11A2* (n = 7; 0.33% of the cohort), both associated with AR/AD forms of NSHL: AD forms of these genes are also described as having postlingual onset. A remarkable finding regarding dominant alleles is that five individuals (0.24%) harbored heterozygous P/LP copy-number variants involving the genes *ABCC1* (n = 2), *TECTA* (n = 1), *DMXL2* (n = 1), and *GSDME* (n = 1).

We also observed a relevant frequency of heterozygotes (n = 262, 12.5%) with recessive alleles for NSHL. This finding is important for genetic counseling, especially for consanguineous couples. AR inheritance represents a relevant fraction of genetic forms of HL and is generally associated with prelingual and more severe clinical involvement (Table 1). *GJB2* was also the gene in which most subjects harbored variants for recessive forms; it is important to note that this gene is associated with both AD and AR inheritances. Biallelic pathogenic variants in *GJB2* are generally described in different populations as the most common genetic cause of sensorineural HL (Azaiez et al., 2004; Kenna et al., 2010). Following this gene is *STRC,* in which we detected two distinct forms of pathogenic CNVs within its coding region. The first form was recurrent and was present in 38 individuals. The second form was nonrecurrent and was found in a single patient. Alongside the CNVs, we detected three P/LP sequence variants, totaling 42 heterozygotes for variants in *STRC.* This gene has also been described as the second most common form of autosomal recessive HL in European populations (Del Castillo et al., 2022).

We used the Hardy–Weinberg equation to estimate the number of affected individuals (homozygotes or compound heterozygotes) with AR forms of HL based on the combined allele frequencies per gene observed in this study and estimated the population frequency of AR NSHR to be 1:2,222 (or 0.45/1,000). This approach presents limitations in certain populations because the frequency of carriers may vary widely in different groups due to founder effects, endogamy, nonrandom mating, and cultural, religious, social, and/or geographical isolation (Antonarakis, 2019).

Fareed et al. (2021) demonstrated how consanguinity influences NSHL occurrence. They found the rare pathogenic *OTOF* (NM_194,248.3): c.2122C>T; p. (Arg708Ter) (rs80356590) in a homozygous state among some individuals from an Indian sample with a consanguineous background. In our cohort, it occurred only once in a male heterozygous individual. His medical records state that he does not have consanguineous parents and we experimentally confirmed this: the sum length of his runs of homozygosity larger than 1 MB (SROH ≥1.0 MB) is approximately 7.0 MB. Matalonga et al., 2020 proposed a minimum of SROH ≥79 MB as evidence of probable consanguinity. In contrast to the sample examined by Fareed et al. (2021), ours was not specifically focused on consanguineous individuals. As such, they constitute a minority (but a relevant portion) of our study (approximately 9.0% or 10.0% depending on the form of assessment).

Recessive forms of NSHL represent an important fraction of congenital deafness, which can be detected by neonatal screening. However, there are other frequent genetic etiologies (such as syndromic forms of HL, *de novo* or inherited AD or XL, and *de novo* or inherited mitochondrial variants) and nongenetic etiologies (congenital infections such as rubella and syphilis; jaundice; and anoxia). Considering this complex etiology, our estimation of 1:2,222 may represent a fraction of congenital HL.

The incidence of congenital HL in Brazil is estimated at 4:1,000 births, with 20% (0.8:1,000 or 1:1,250) of cases having a genetic etiology (Schüffner et al., 2020). Interestingly, our estimate (1:2,222) is not very far from the epidemiological study.

We did not find any P/LP variants in the X chromosome or mitochondrial DNA. We believe that the primary reason for this is that both XL and mitochondrial inheritance are indeed uncommon causes of NSHL (Shearer and Smith, 2015), accounting for approximately 2–5% and 1% of all cases, respectively, and we would expect very low frequencies or even the absence of these variants in a cohort of unaffected individuals, such as ours. However, many HL studies do not even investigate the X chromosome or the mitochondrial genome, and both likely harbor variants yet to be found.

All 2,097 patients analyzed for statistics and population assumptions in this study were not affected with HL, as we eliminated all 89 patients with HL phenotypes, but they are not true “controls,” since they comprise a convenience sample referred by participating research centers from Brazil to undergo genome sequencing because they presented clinical manifestations (other than hearing impairment) for rare diseases suspected of genetic etiology. This fact may explain the higher consanguinity frequency in our cohort. Although we acknowledge that the ideal group of patients for the purpose of our study and for more precise population assumptions would comprise asymptomatic adult individuals, we compared the frequencies of variants found in this study to the frequencies found in the ABraOM repository, which contains genomic variants from elderly individuals from Brazil, and did not find relevant differences for the great majority of variants co-occurring in both databases (Supplementary Table S4). This may suggest that despite our sample being enriched with individuals with rare diseases, the frequencies for HL-associated pathogenic variants in our sample of individuals without HL may be representative of the frequency in the general Brazilian population as well.

The approach of using groups of symptomatic individuals with heterogeneous conditions as controls has been widely used before, such as in several studies that used molecular data from the United States National Institutes of Health (NIH) Heart, Lung and Blood Institute (NHLBI)-sponsored Exome Sequencing Project, composed primarily of symptomatic individuals, as control groups (Fu et al., 2013). In this study, we used the same strategy of examining datasets of people with diverse symptomatic conditions and without HL as a valid approach for use as controls for HL.

Gene selection for common genetic conditions that multiple genes are capable of producing the same phenotype, such as NSHL, may be a challenging task. Our first step in preselecting genes associated with a diverse group of genetic disorders was very inclusive and comprised several genes with limited evidence of gene-disease association, most of which presented few clinical reports and/or limited functional studies, and some genes with contradictory evidence of the association with HL. As we did not find a previously published systematic approach for gene-disease classification for NSHL, we applied an existing framework proposed by ClinGen and observed that half (21/42) of the genes were associated with AD NSHL, and more than one-third (23/65) of AR genes, half (3/6) of X-linked genes, and one-fourth (4/16) of all forms of the eight AR/AD genes exhibited limited gene-disease association. A limited gene-disease validity does not necessarily delegitimize studies with this group of genes, but it implies that the literature remains limited either in the number of individuals described or experimental data (Strande et al., 2017). Therefore, these results and conclusions regarding genes with limited association with diseases must be interpreted with caution.

An accurate clinical validity of gene-disease association is likely the first important step in understanding the genomics of NSHL. The second step is understanding the clinical impact of the variants harbored by these genes. Therefore, we carefully reviewed the available evidence and eliminated genes that were only weakly associated with NSHL: some genes were removed from the analysis either because their association with NSHL was classified as disputed or refuted (such as the AR NSHL-associated variants of *GJB6*). Among the disputed genes is *GJB3*. One relevant variant in *GJB3* (NM_024009.3: c.196_198del; p.Asp66del) was quite frequent, with six heterozygotes (AF = 0.0014), but was eliminated from our analysis because we considered this gene not to exhibit a minimal level of gene-disease association.

Another challenging task in this study was selecting relevant variants associated with HL because, on the one hand, exaggeratedly inclusive approaches may overestimate frequencies, especially when including variants by presumptively assuming a deleterious mechanism without proper functional studies or relevant reports by other researchers. On the other hand, restricted inclusions may underestimate frequencies, especially for hypomorphic alleles (such as missense variants) that do not result in clear loss of function or variants not previously reported in the literature or genomic databases.

Both the aforementioned approaches represent relevant biases and may influence correct population assumptions, especially for rare molecular mechanisms and genes with limited evidence for gene-disease association. As a means to balance this conflict, we chose an approach that restricted the selection of variants for those already observed and classified as P/LP by at least one participant in the ClinVar database, which is the largest and most used public archive of reports of the relationships among human variations and phenotypes from participating centers from the whole world (Landrum et al., 2018). These preselected variants were then curated by our team of researchers and strictly reclassified following the ACMG criteria for variant classification. The direct consequence of this approach, though, is that we have lost all variants not present in the ClinVar database.

In our previous study, we used exome sequencing to search for the carrier status for AR conditions, including AR forms of HL, in a cohort of 320 patients (Quaio et al., 2021). We found a total of 27 occurrences of P/LP variants associated with either syndromic or NSHL. Among these variants, five (19.5%) were not found in the ClinVar database: two were harbored by genes with definite association with NSHL (*DIAPH1* and *GIPC3*), one was harbored by a gene with strong association (*MYO3A*), one had limited association (*TBC1D24*), and another one was associated with a syndromic form of HL (*PHYH*). This suggests that we may have lost approximately 15% of potentially relevant variants harbored by genes associated with HL in the current study, as we restricted the scope of variants solely to those present in ClinVar.

Large-scale genomic studies of NSHL in non-North American and non-European populations are limited in the literature. We did not find comprehensive studies in Latin America for robust comparisons to our results. We hope that our pioneer study stimulates other groups in future attempts to better understand the molecular events associated with NSHL, especially those from countries with underrepresented populations in genomic studies.

In conclusion, we collected whole-genome sequencing data from a cohort of 2,097 Brazilian individuals unaffected by HL to investigate the molecular findings associated with NSHL and determined relevant frequencies of molecular alterations (sequence variants and CNVs) associated with this group of conditions and estimated a combined frequency of all recessive forms of NSHL of one case per 2,222 individuals. This approach demonstrates the potential for determining clinical and social burdens at the population level and addresses health policies, confirming the clinical utility of obtaining genomic data for the investigation and management of hearing impairment.

## Data Availability

The original contributions presented in the study are included in the article/Supplementary Material, further inquiries can be directed to the corresponding authors.
